# Continuation rate for asenapine and brexpiprazole treatment in patients with schizophrenia

**DOI:** 10.1002/brb3.2109

**Published:** 2021-03-13

**Authors:** Yuichi Inoue, Hidenobu Suzuki, Hiroyuki Hibino, Atsuhiko Takaya, Katsunaka Mikami, Kenji Yamamoto, Hideo Matsumoto

**Affiliations:** ^1^ Department of Psychiatry Shakomae Kokorono Clinic Tokyo Japan; ^2^ Department of Psychiatry Suzuki Clinic Tokyo Japan; ^3^ Department of Psychiatry Fukui Kinen Hospital Kanagawa Japan; ^4^ Department of Psychiatry Course of Specialized Clinical Science Tokai University School of Medicine Kanagawa Japan

**Keywords:** asenapine, brexpiprazole, drug discontinuation, influencing factors, schizophrenia

## Abstract

**Introduction:**

The current study sought to compare the treatment continuation rates of asenapine and brexpiprazole while specifically investigating the factors influencing this index and the clinical efficacy of brexpiprazole.

**Methods:**

Retrospective study on patients with schizophrenia who were prescribed either asenapine (*n* = 73) or brexpiprazole (*n* = 136), as part of their routine medical care.

**Results:**

The treatment continuation rates for asenapine and brexpiprazole were 19.0% and 38.6% at 52 weeks, with that of brexpiprazole found to be significantly higher than that of asenapine (*p* = .002). Moreover, age was found to be a significant factor affecting the treatment continuation rate for brexpiprazole (*p* = .03). Additionally, patients with a longer continuation duration had significantly lower Clinical Global Impression‐Severity of Illness (CGI‐S) scale scores compared to those who discontinued early (*p* = .04). The continuation rate was also significantly higher for those who began using the drug as outpatients compared to those first administered the drug as inpatients (*p* = .04). Furthermore, disease duration, CGI‐S scale, and continuation duration significantly affected the clinical efficacy of brexipiprazole (*p* < .05 for all).

**Conclusions:**

The continuation rate for brexpiprazole increases as the age of the patient increases, as disease severity decreases, and if the patient first uses the drug as an outpatient. Shorter disease duration and longer drug administration may lead to improved clinical efficacy. These results suggest that brexpiprazole is an effective treatment option for maintenance therapy of schizophrenia.

## Graphical abstract

This retrospective study sought to compare the treatment continuation rates of asenapine and brexpiprazole while examining the specific factors that affect both the continuation rates and clinical efficacy of brexpiprazole in patients with schizophrenia. Results show that brexpiprazole had a significantly higher treatment continuation rate compared to asenapine. Moreover, patients who were older, had a lower CGI‐severity of illness score, and those for whom the drug was initiated as an outpatient, had a higher continuation rate. Meanwhile, disease duration, CGI‐S scale, and continuation duration all significantly affected the clinical efficacy of brexpiprazole.

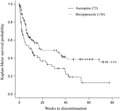

## 1 INTRODUCTION

Schizophrenia is a chronic disease requiring long‐term antipsychotic therapy from the acute phase through to remission. However, nonadherence and partial nonadherence to antipsychotic therapy commonly occur among patients with schizophrenia. In fact, continuous daily oral consumption of medication has been reported as a challenge for over half of patients with schizophrenia (Valenstein et al., 2006; Weiden et al., 2004). Moreover, considering that the risk of recurrence and hospitalization increases as a result of nonadherence (Morken et al., 2008), it is imperative to select drugs that strike a balance between clinical efficacy and adverse effects, to ensure a sufficiently high level of drug adherence.

One method for assessing drug adherence is application of the treatment continuation rate as an index. Clearly, drugs with a high treatment continuation rate in antipsychotic therapy are desirable; therefore, obtaining data regarding whether a drug is suitable for a particular patient, as well as data that provides insights into the factors that affect the treatment continuation rate and clinical efficacy of a drug, is important elements that serve to inform appropriate drug selection.

Since the 1996 approval of risperidone in Japan, several second‐generation antipsychotic medications have been introduced for clinical use; currently, the core drugs used for this purpose are schizophrenia drugs. Specifically, asenapine was introduced for clinical use in 2016, while brexpiprazole was not introduced until 2018.

Asenapine has a high affinity for a wide range of receptors, including those for dopamine D_2_, serotonin, α_1_ adrenaline, and histamine, while exhibiting low affinity for the muscarinic acetylcholine receptor (Shahid et al., 2009). Additionally, asenapine is administered in the form of a sublingual tablet, which has not been previously applied to antipsychotic drugs. Alternatively, brexpiprazole has an intrinsic activity as a dopamine D_2_ receptor partial agonist and is believed to exhibit powerful partial agonist activity on the serotonin 5‐HT_1A_ receptor while also serving as a 5‐HT_2A_ receptor antagonist. Hence, brexpiprazole is referred to as a “serotonin‐dopamine activity modulator” (SDAM) (Maeda, Lerdrup, et al., 2014; Maeda, Sugino, et al., 2014). Asenapine and brexpiprazole, therefore, have differing receptor affinities compared to those of previous antipsychotic drugs, yet offer the same efficacy, safety, and tolerance as other second‐generation antipsychotic drugs (Correll et al., 2016; Kane et al., 2016; Leucht et al., 2013). Accordingly, asenapine and brexpiprazole are considered new schizophrenia drug therapy options that are predicted to be as, or more, suitable for long‐term use in schizophrenic patients than other second‐generation antipsychotics. On the other hand, in a previous study in Japan, Azekawa et al., (2011) have already compared the continuation rates of oral second‐generation antipsychotics, and we have also compared the continuation rates of second‐generation antipsychotics long‐acting injections (Suzuki et al., 2018). However, few studies have investigated the treatment continuation rates of asenapine and brexiprazole. In addition, because brexpiprazole is the newest drug to be introduced for clinical use in Japan, there have been no studies investigating the factors that affect its treatment continuation rate or clinical efficacy.

Therefore, in the present study, we conducted a retrospective survey of patients with schizophrenia who were prescribed asenapine or brexpiprazole as part of their routine medical care. We then compared the treatment continuation rates of both drugs, with a particular focus on investigating the factors that affect the continuation rates and clinical efficacy of brexpiprazole, the most recently introduced drug in Japan.

## 2 METHODS

### 2.1 Patients and study design

The participants enrolled in this retrospective study were out‐ and inpatients at Shakomae Kokorono Clinic and Fukui Kinen Hospital. All patients were diagnosed with schizophrenia according to the diagnostic criteria provided in the Diagnostic and Statistical Manual of Mental Disorders, 5th edition (DSM‐V; American Psychiatric Association, 2013) and were prescribed either asenapine or brexpiprazole. The observation period was from May 2016 (when introduced for clinical use) to 1 January 2018 for asenapine and April 2018 (when introduced for clinical use) to 1 December 2019 for brexpiprazole. We also investigated the impact of specific brexpiprazole treatment continuation rate and efficacy, including age at the time of initiating drug administration, sex, disease duration, number of hospitalizations prior to brexpiprazole use, drug administration started as outpatient or inpatient, Clinical Global Impression‐Severity of Illness (CGI‐S) and Improvement (CGI‐I) scale scores, and concurrent use of other antipsychotic drugs (yes vs. no).

In addition, the cessation of drug administration was considered an “event,” the period up to the occurrence of the “event,” “closure,” or “conclusion of observation” was included in the observation period used in our statistical analysis. The reasons for the “event” occurrence were categorized using the Clinical Antipsychotic Trial of Intervention Effectiveness (CATIE) study (Lieberman et al.; 2005) as “for lack of efficacy,” “owing to intolerability,” “death,” “owing to patient's decision,” or “for other reasons”. This study was approved by the ethics committee of Fukui Kinen Hospital.

### 2.2 Statistical analysis

Comparison of patient background characteristics (sex, inpatient/outpatient status at initiation, use of other antipsychotic drugs) was conducted using the chi‐square test, while the Mann–Whitney *U* test was used to compare age, disease duration (years), number of hospitalizations prior to the start of drug administration, CGI‐S, and CGI‐I scores. The treatment continuation rate was estimated using Kaplan–Meier survival analysis. Comparisons of the continuation rates were performed using the log‐rank test, Fisher's exact test was applied to determine whether there was a difference in the classification distribution for the discontinuation (cessation) reasons. We used the log‐rank test to determine whether there was a difference in the treatment continuation rate in term of the number of weeks until discontinuation and the three common reasons for discontinuation as observed in this study “owing to patient's decision,” “owing to intolerability,” and “for lack of efficacy.”

Next, to identify factors that had an effect on the brexpiprazole treatment continuation rate, we used the Cox proportional hazards regression model with the brexpiprazole continuation duration as the dependent variable and age, sex, disease duration, CGI‐S score, number of hospitalizations until the start of drug administration, and status at the start of drug administration, as the independent variables. We further divided the brexpiprazole continuation duration into a “Continuation for 24 weeks or more” group and a “Discontinuation in under 24 weeks” group, and subsequently performed logistic regression analysis using these groups as the response variables, and age, sex, status at start, and CGI‐S score as the predictor variables. Our investigation of the differences between the two groups using the factors in each group consisted of the following: chi‐square test was performed for sex; Mann–Whitney *U* test for age, disease duration (years), number of hospitalizations prior to the start of drug administration, and CGI‐S score, Fisher's exact test for status at the start of drug administration and concurrent use of other antipsychotic drugs (yes vs. no). To identify factors that affect the clinical efficacy of brexpiprazole, we created a “CGI‐I 3 and under” group and a “CGI‐I 4 and above” group and performed logistic regression analysis using the above two groups as the response variables and age, sex, disease duration, CGI‐S score, and continuation duration (weeks) as the predictor variables. All statistical analyses were performed with EZR (Kanda, 2013), which was used for R. More precisely, it is a modified version of R commander designed to add statistical functions frequently used in biostatistics. The significance level was *p* < .05.

## 3 RESULTS

### 3.1 Patient characteristics

The patient details obtained from the survey were as follows: 73 patients were prescribed asenapine, and 136 were prescribed brexpiprazole (total patients = 209). No significant differences were observed for any of the patient characteristics between the two groups (Table 1). In addition, no patients taking asenapine and brexpiprazole concurrently were included in the study.

**TABLE 1:** Patient characteristics

Characteristic	ASP (*n* = 73)	BRX (*n* = 136)	*p* value
Sex (Male/Female)	25/48	60/76	.27^a^
Mean age at start of study ± *SD*, years	47.1 ± 13.2	49.9 ± 15.2	.22^b^
Mean during the illness ± *SD*, years	20.8 ± 12.1	20.4 ± 13.7	.77^b^
Inpatient/outpatient status at initiation	59/14	106/30	.76^a^
Number of hospitalizations before the start of study ± *SD*	4.9 ± 4.5	3.9 ± 3.5	.13^b^
Clinical Global Impression‐Severity of illness ± *SD*	5.0 ± 1.0	4.8 ± 0.9	.17^b^
Concurrent use of other antipsychotic drugs (yes/no)	59/14	107/29	.85^a^
Clinical Global Impression‐Improvement ± *SD*	3.8 ± 0.4	3.5 ± 0.9	.46^b^

^a^ Analyzed using the χ^2^ test

^b^ Analyzed using the Mann–Whitney *U* test

### 3.2 Treatment continuation rate

**FIGURE 1:**
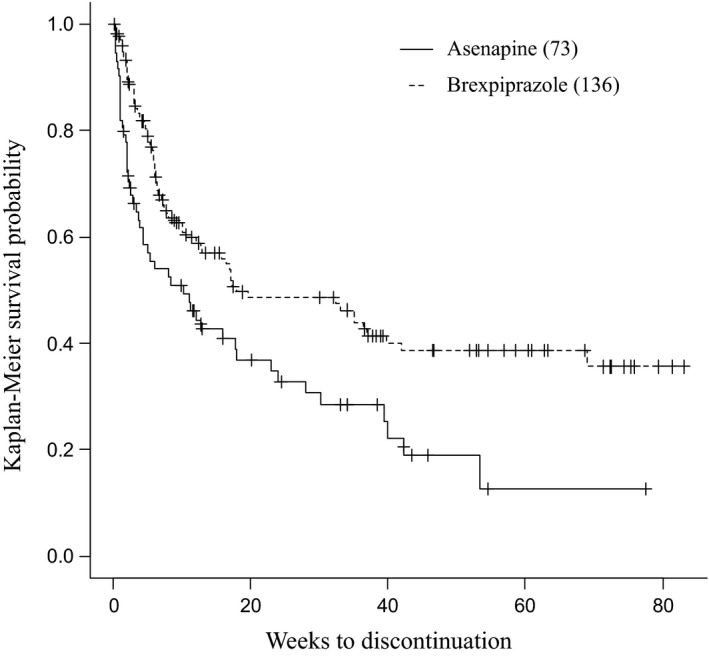
Kaplan–Meier analysis of time to discontinuation of asenapine and brexpiprazole

The treatment continuation rates for asenapine and brexpiprazole were 32.8% and 48.5% at 24 weeks, respectively; and 19.0% and 38.6% at 52 weeks, respectively. These data suggest that brexpiprazole had a significantly higher treatment continuation rate than asenapine (*p* = .002; Figure 1).

### 3.3 Reasons for discontinuation

**FIGURE 2:**
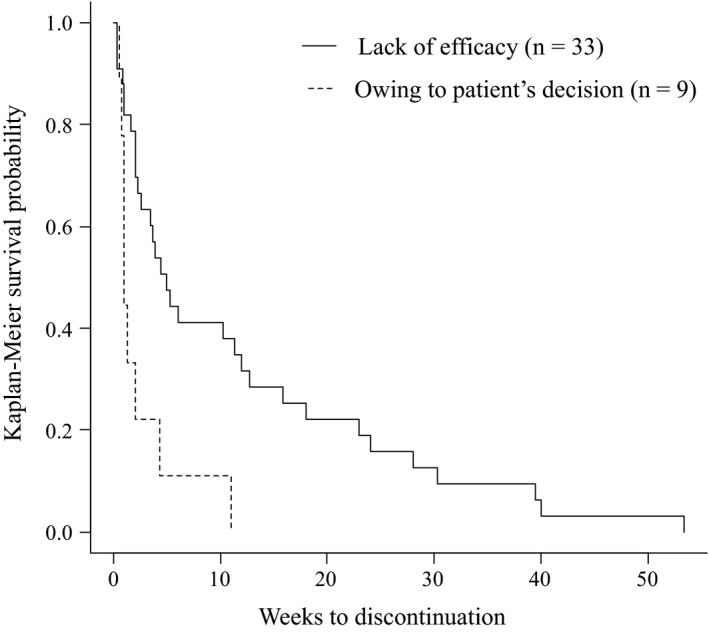
Duration of asenapine continuation until discontinuation due to “Owing to patient's decision” and “For lack of efficacy”

The main reasons for discontinuation of asenapine were lack of efficacy (67% of discontinuers), owing to patient's decision (18% of discontinuers), and owing to intolerability (10% of discontinuers). On the other hand, the main reasons for discontinuation of brexpiprazole were lack of efficacy (80% of discontinuers), owing to intolerability (10% of discontinuers), and owing to patient's decision (6% of discontinuers). Therefore, there were no significant differences between the number of patients in the asenapine and brexpiprazole groups who discontinued treatment for any of the reasons investigated in this study. However, the duration of asenapine use until discontinuation due to “owing to patient's decision” was significantly shorter than that due to “for lack of efficacy” (*p* = .003; Figure 2). Moreover, six cases of asenapine and seven cases of brexpiprazole were discontinued due to side effects. For asenapine, these included drowsiness in four cases, aspiration pneumonia in one case, and lameness in one case. Meanwhile, the side effects of brexpiprazole were leukopenia in one case, malignant syndrome in two cases, extrapyramidal symptoms in one case, QTc prolongation in one case, fever in one case, and dysphagia in one case.

### 3.4 Factors that affect brexpiprazole treatment continuation rate

Using the Cox proportional hazards regression model, age was determined to significantly affect brexpiprazole continuation duration (*p* = .032; Table 2). We also determined the factors that affect brexpiprazole continuation duration based on the comparison between the “Continuation for 24 weeks or more” and “Discontinuation in under 24 weeks” groups. We again determined, via logistic regression analysis, that age significantly affected brexpiprazole continuation duration (*p* = .039). Meanwhile, based on patient characteristics, the continuation group had significantly lower pre‐brexpiprazole CGI‐S scores compared to the discontinuation group (*p* = .039; Table 3). Additionally, those who initiated drug administration as outpatients had significantly higher continuation rates than those who started as inpatients (*p* = .043).

**TABLE 2:** Factors that affect the brexpiprazole continuation duration as identified by the Cox proportional hazards regression model

Factor	Hazard ratio	95% CI		*p* value
		Lower	Upper	
Age	0.98	0.96	1.00	.032
Sex (Male/Female)	1.30	0.80	2.10	.29
Duration of illness	1.01	0.98	1.03	.72
Clinical Global Impression‐Severity of illness	1.22	0.90	1.65	.20
Number of hospitalizations	1.03	0.95	1.11	.47
Patients status (in‐out)	1.35	0.65	2.82	.42

**TABLE 3:** Factors that affect the brexpiprazole continuation duration based on patient characteristics

Factor	Continuation for 24 weeks or more (*n* = 44)	Discontinuation in under 24 weeks (*n* = 61)	*p* value
Sex ( Male/Female )	20/24	29/32	.990^a^
Age (years) (Mean ± *SD*)	54.9 ± 14.8	49.2 ± 14.9	.075^b^
Duration of illness (years) (Mean ± *SD*)	21.8 ± 13.9	22.1 ± 13.5	.810^b^
Patients status (in‐out)	16/28	11/50	.043^c^
Number of hospitalizations (Mean ± *SD*)	4.2 ± 4.4	4.2 ± 3.1	.267^b^
Clinical Global Impression‐Severity of illness, (Mean ± *SD*)	4.6 ± 1.1	5.0 ± 0.9	.039^b^
Concurrent use of other antipsychotic drugs (yes/no)	36/8	51/10	.800^c^

^a^ Analyzed using the χ^2^ test.

^b^ Analyzed using the Mann–Whitney *U* test.

^c^ Analyzed by Fisher's exact test.

### 3.5 Factors that affect the clinical efficacy of brexpiprazole

We found that disease duration, CGI‐S score, and treatment continuation duration, all significantly affected the clinical efficacy of brexpiprazole (*p* = .019, .010, and .009, respectively. Table 4).

**TABLE 4:** Factors impacting the clinical efficacy of brexpiprazole

Factor	Odds ratio	95% CI		*p* value
		Lower	Upper	
Age	0.99	0.96	1.02	.650
Sex (Male/Female)	0.53	0.24	1.16	.110
Duration of illness	0.96	0.93	0.99	.019
Clinical Global Impression‐Severity of illness	0.55	0.35	0.87	.010
Continuation duration	1.02	1.01	1.04	.009

## 4 DISCUSSION

The primary objective of this study was to conduct a comparison between the treatment continuation rates of asenapine and brexpiprazole. This index can be influenced by various factors, including physician‐patient relationship, drug efficacy, safety, tolerability, and drug preference. In the present study, no significant differences were observed between the two drugs in terms of CGI‐I scores; however, brexpiprazole was found to have a significantly higher treatment continuation rate than asenapine. In a previous study, the retention rate for asenapine after 52 weeks was 38% (Schoemaker et al., 2010), which was higher than that in the present study; whereas the retention rate for brexpiprazole was 43% (Forbes et al., 2018), which was similar to that in the present study.

A possible reason for the superior continuation rate of brexpiprazole may be related to drug preference. Although we did not evaluate drug preference in this study, we did observe that the rates of discontinuation due to “owing to patient's decision” were higher for asenapine than for brexpiprazole. In addition, the duration until discontinuation of asenapine due to “owing to patient's decision” was significantly shorter than that observed due to “for lack of efficacy.” Discontinuation as a result of "owing to patient's decision" was reported as due to the bitter taste and oral discomfort when taking asenapine. Alternatively, the duration to discontinuation of brexpiprazole due to "owing to patient's decision" was not significantly different from that due to "for lack of efficacy" or "owing to intolerability." Furthermore, the time to discontinuation of asenapine for the reason of "owing to patient decision" was not significantly different from the time to discontinuation of brexpiprazole for the same reason. In this study, we were not able to determine the factors affecting the difference in treatment continuation rates between asenapine and brexpiprazole based on the reasons for discontinuation.

The results of a previous study suggested that the most commonly experienced adverse effects of asenapine were drowsiness and sedation, and that these effects occur shortly after initiating drug administration (Citrome, 2014). It has also been reported that the sublingual tablet form of asenapine may cause oral hypoesthesia and dysgeusia to occur at a frequency of approximately 5% (Citrome, 2013). Further, it was previously reported that the frequency of adverse effects associated with brexpiprazole, such as sleepiness, nausea, and insomnia, did not differ from that of the placebo (Kane et al., 2016).

Additionally, aripiprazole, a drug used prior to the introduction of brexpiprazole, is clinically effective and has been previously suggested to be highly accepted by patients (Tandon et al., 2006). However, there is a need to conduct further investigation into the factors that affect the difference between the treatment continuation rates of the two drugs.

The secondary objective of this study was to investigate the factors that affect the treatment continuation rate and clinical efficacy of brexpiprazole. Specifically, the results of this investigation suggest the possibility that the brexpiprazole treatment continuation rate may be improved when the age of the patient is higher and by initiating the drug therapy on an outpatient basis. Further, lower degrees of disease severity may also increase the treatment continuation rate of brexpiprazole.

Similarly, in their 3‐year prospective follow‐up study of risperidone long‐acting injection (RLAI) therapy, Taylor et al., (2009) reported that the discontinuation rate was higher among “young (patients),” when there was a “long disease duration,” and when drug administration was initiated when the patient had “in‐patient status,” as well as when the “RLAI dose was 25 mg/2 weeks.” Their investigation into the possible reasons for this indicates that “young (patients),” “long disease duration,” and “in‐patient status” were factors that indicated worsening illness, which led them to postulate that no matter what antipsychotic drug was utilized, the prognoses of patients were poor. It has also been reported that elderly patients tend to have a strong preference for maintenance of their current condition (Fraenkel et al., 2015). Hence, elderly patients may be more open to the use of antipsychotic drugs owing to the possibility that they had previously tried several different drugs (Kreyenbuhl et al., 2011). These factors might have also influenced the treatment continuation rate in the current study.

Alternatively, previous research results suggest that high doses of antipsychotic drugs taken prior to the start of brexpiprazole administration may increase the risk of discontinuing brexpiprazole (Yoshimura et al.,^2020^). Nevertheless, few studies have investigated the factors that affect the treatment continuation rate of brexpiprazole, further investigation on this issue is required.

Next, the results of our investigation on the factors that affect the clinical efficacy of brexpiprazole suggest that clinical efficacy may be improved when the disease duration is shorter and drug administration period is longer. Similarly, Takase et al., (2015) found that the group of patients who had developed dopamine‐sensitive psychosis prior to switching to aripiprazole had significantly higher doses of antipsychotics prior to the introduction of aripiprazole and a significantly higher rate of discontinuation due to worsening psychosis compared to the group of patients who had not developed dopamine‐sensitive psychosis. In addition, 8% of patients who did not have dopamine‐sensitive psychosis experienced worsening during the switching process to aripiprazole, however, this group of patients had significantly higher prior antipsychotic doses, comparable to those in the group of patients who experienced dopamine‐sensitive psychosis, compared to other patients who did not have dopamine‐sensitive psychosis. In the aforementioned study by Yoshimura et al., (2020), it was suggested that high doses of antipsychotic medication prior to induction may increase the risk of discontinuation, even with brexpiprazole. Although we did not investigate the dosage of antipsychotic medication prior to induction in the current study, the short duration of illness indicates that antipsychotic dosage was not increased due to repeated symptom relapses, and thus, the risk of developing dopamine‐sensitive psychosis is low. This may also indicate that the longer duration of brexpiprazole treatment affected the clinical efficacy of the study. As there have been few studies on the factors that affect the clinical efficacy of brexpiprazole, further investigation is warranted.

Considering the results of this study, brexpiprazole may represent one of the best treatment options for schizophrenia maintenance therapy. This study also identified the possibility that the treatment continuation rate of brexpiprazole may improve when the disease severity is low, when brexpiprazole administration is initiated on an outpatient basis, and with advanced patient age. Furthermore, we suggest that the clinical efficacy of brexpiprazole may improve when the disease duration is short and the drug administration period is long. As mentioned earlier, the onset of dopamine‐hypersensitivity psychosis has been suggested to shorten treatment retention, the shorter the duration of illness, the greater the drug response, and furthermore, the less dopamine receptors are over‐blocked by antipsychotics, the lower the risk of developing dopamine‐hypersensitivity psychosis, suggesting that the duration of illness may guide clinicians' choice of the dopamine partial agonist brexpiprazole.

Since this study was a retrospective survey that recorded real clinical outcomes, there are several factors to consider when interpreting the results. First, because the study included all patients treated with either asenapine or brexpiprazole at each institution where data were collected, the number of patients treated with asenapine was smaller than that treated with brexpiprazole. Second, the number of sites where the study was conducted was small, and the selection of subjects for drug treatment, as well as for the continuing treatment index, was based on judgments of efficacy, tolerability, etc. by the therapists, without using the psychiatric symptom rating scale or the side effect rating scale. Therefore, selection bias may have occurred, potentially causing bias in the outcome variable analysis. Third, since we did not focus on monotherapy but rather included cases of combination pharmacotherapy, we cannot fully rule out the possibility that drugs being concurrently administered may have affected the findings. Therefore, to confirm our findings, prospective monotherapy studies with rating scales are needed.

## 5 CONCLUSIONS

The continuation rate for brexpiprazole increases as the age of the patient increases, as disease severity decreases, and if the patient first uses the drug as an outpatient. Shorter disease duration and longer drug administration may lead to improved clinical efficacy. These results suggest that brexpiprazole is an effective treatment option for maintenance therapy of schizophrenia.

## CONFLICT OF INTEREST

Dr. Inoue has received honoraria from Janssen Pharmaceutical K.K and Otsuka Pharmaceutical Co., Ltd. Dr. Suzuki has received honoraria from Janssen Pharmaceutical K.K, Otsuka Pharmaceutical Co., Ltd, Meiji Seika Pharma Co., Ltd, and MSD K.K. Dr. Hibino has received honoraria from Janssen Pharmaceutical K.K, Otsuka Pharmaceutical Co., Ltd, and Dainippon Sumitomo Pharma Co., Ltd. Dr. Takaya has received honoraria from Otsuka Pharmaceutical Co., Ltd, Daiichi Sankyo Co., Ltd, and Eli Lily and Company. Dr. Mikami received: a) research supports from Grant‐in Aid for Scientific Research (C) (Grant Number 18K07611), Taisho Pharmaceutical, Otsuka Pharmaceutical, Shionogi & Co., Japanese Society for Probiotic Science; b) honoraria from Otsuka Pharmaceutical, Shionogi & Co., Shire Japan, Eli Lilly and Co., Meiji Holdings Co., Takeda Pharmaceutical, Miyarisan Pharmaceutical Co; and c) a consulting fee from Otsuka Pharmaceutical and Shionogi & co. Dr. Yamamoto reports receiving grants and personal fees from Eisai Co., Ltd., Japan; grants and personal fees from Otsuka Pharmaceutical Co., Ltd., Japan; personal fees from Meiji Seika Pharma Co., Ltd.; personal fees from Dainippon Sumitomo Pharma Co., Ltd.; personal fees from Pfizer Japan Inc.; personal fees from Mitsubishi Tanabe Pharma Corporation; personal fees from Shionogi & Co.; personal fees from Eli Lilly and Company; personal fees from EPS Holdings, Inc.; grants from Grant‐in‐Aid for Scientific Research (16K 10260) outside the submitted work. Dr. Matsumoto received: a) research support from Dainippon Sumitomo Pharma Co., Ltd.; Otsuka Pharmaceutical Co., Ltd.; Eli Lilly and Company; and Mitsubishi Tanabe Pharma Corporation, Shionogi & Co., Ltd.; b) honoraria from Eli Lilly and Company; Novartis Pharma K.K.; Yoshitomiyakuhin Corporation; GlaxoSmithKline; Dainippon Sumitomo Pharma Co., Ltd.; Pfizer Inc.; Meiji Seika Pharma Co., Ltd.; Otsuka Pharmaceutical Co., Ltd.; Janssen Pharmaceutical K.K.; Eisai Co. Ltd.; Shionogi & Co., Ltd; Astellas Pharma Inc.; and Mitsubishi Tanabe Pharma Corporation, MSD K.K.

## AUTHOR CONTRIBUTION

Yuichi Inoue contributed to design and conceptualization of the study, acquisition/analysis/interpretation of the data, and drafting/revising the manuscript. Hidenobu Suzuki contributed to drafting/revising the manuscript. Hiroyuki Hibino contributed to design and conceptualization of the study, acquisition/analysis/interpretation of the data. Yuichi Inoue, Hidenobu Suzuki, and Hiroyuki Hibino contributed equally to the manuscript. Atsuhiko Takaya contributed to acquisition/interpretation of the data. Katsunaka Mikami, Kenji Yamamoto, and Hideo Matsumoto supervised the study. All authors discussed the results and reviewed the manuscript.

### PEER REVIEW

The peer review history for this article is available at https://publons.com/publon/10.1002/brb3.2109.

[Correction added on March 20, 2021, after first online publication: Peer review history statement has been added.]

## Data Availability

The data that support the findings of this study are available from the corresponding author upon reasonable request.
